# In Vitro Determination of Cytotoxic Effects of Ten Essential Oils on *Prototheca bovis,* Which Causes Mastitis in Dairy Cows

**DOI:** 10.3390/ijms26125451

**Published:** 2025-06-06

**Authors:** Maria Kuczyńska, Magdalena Kot, Marcin Stocki, Ewa Zapora, Tomasz Jagielski, Magdalena Perlińska-Teresiak, Aleksandra Kalińska

**Affiliations:** 1 Department of Animal Breeding, Institute of Animal Sciences, Warsaw University of Life Sciences, 02-787 Warsaw, Poland; s207313@sggw.edu.pl (M.K.); magdalena_kot@sggw.edu.pl (M.K.); 2 Department of Silviculture and Forest Utilization, Institute of Forest Sciences, Bialystok University of Technology, Wiejska 45A Str, 15-351 Bialystok, Poland; m.stocki@pb.edu.pl (M.S.); e.zapora@pb.edu.pl (E.Z.); 3 Department of Medical Microbiology, Institute of Microbiology, Faculty of Biology, University of Warsaw, 02-096 Warsaw, Poland; t.jagielski@uw.edu.pl; 4 Department of Animal Genetics and Conservation, Institute of Animal Sciences, Warsaw University of Life Sciences, 02-787 Warsaw, Poland; magdalena_perlinska_teresiak@sggw.edu.pl

**Keywords:** microalgae, essential oils, plant extracts, udder inflammation, mastitis pathogens, antibiotic resistance

## Abstract

Mastitis is a common condition in dairy cattle that causes huge losses globally. The inflammation is caused by the invasion of the teat canal by pathogens, including hard-to-control single-cell microalgae of the genus *Prototheca*. The aim of the study was the in vitro comparison of the antimicrobial properties of 10 selected essential oils (EOs) and amphotericin B (AMB) against *Prototheca bovis* strains (PRO3 and PRO7) from different regions in Poland. The antialgal effect was estimated by using toxicity tests. The chemical composition of the EOs was determined by using gas chromatography coupled with mass spectrometry. The tested EOs had significant cytotoxic effects on algal viability. A statistical analysis of the results revealed that the highest biocidal potential, at a concentration of 2%, was demonstrated by lavender, rosemary, and oregano oils, reducing the survival of the *Prototheca bovis* strains, on average, by 51.21%, 45.83%, and 45.15%, respectively. In comparison, AMB reduced algal viability by an average of 88% compared with the control groups. Further research into the utilization of the biocidal properties of lavender, rosemary, and oregano oil against *Prototheca* spp. may help to develop new forms of treatments against mastitis caused by this pathogen in the future.

## 1. Introduction

Mastitis is a problem affecting the vast majority of dairy herds, despite the fact that appropriate hygiene rules are followed by farmers [1]. The inflammation, which can be clinical or subclinical, is mainly caused by the invasion of the teat by pathogens that include bacteria, fungi, viruses, and algae. Factors that favor the development of mastitis include the aging of the cow, a high level of exploitation, and an ongoing drying-out period [2]. The occurrence of clinical mastitis is less frequent than subclinical mastitis and is easier to detect due to its characteristic symptoms, such as the swelling of the udder and the induration or soreness of the quarters. In addition, the characteristics of the milk are altered: it becomes waterier, clots appear, and it may also have a different color [1]. The subclinical form, on the other hand, may not show any symptoms, apart from a significantly increased somatic cell count (SCC) in the milk and a decrease in the milk yield of affected cows. The SCC in the milk should not exceed 200,000/cm^3^; otherwise, the presence of inflammation is indicated [3]. The latent and chronic course of subclinical mastitis makes it much more difficult to control and treat; it, therefore, contributes to greater financial losses [1]. Costs are incurred, to the greatest extent, by factors such as loss of milk production, medical costs, discarded milk, and excess labor demand, as well as the premature culling of animals [4]. Among the bacterial pathogens contributing to mastitis, the most common are streptococci, including *Staphylococcus aureus*, *Escherichia coli*, *Klebsiella* spp., *Trueperella pyogenes*, *Pseudomonas* spp., and less frequently *Corynebacterium* spp. and *Mycobacterium* spp. In the case of mastitis caused by fungi, the most commonly isolated genera are *Trichosporon, Rhodotorula*, *Cryptococcus*, and especially *Candida* spp. [5]. There are also an increasing number of cases of infections caused by unicellular algae of the genus *Prototheca*. Numerous reports of rising case numbers have emerged from countries such as China, Italy, Korea, Japan, Brazil, and Poland [1,6,7,8,9].

*Prototheca* spp. are the only algae known so far to have pathogenic potential for both animals and humans [10]. Algae of the genus *Prototheca* were classified as mastitis-causing pathogens relatively recently—the first case of infection was documented in 1952 [11]. Prior to that, the pathogen was regarded as an insignificant saprophytic microalgae or was misidentified as a species belonging to yeast. Currently, it is known that *Prototheca* spp. and, in particular, the species *Prototheca blaschkeae*, *Prototheca wickerhamii*, and *Protothca bovis* (former *Prototheca zopfii* subtype II) [12] represent the cause of mastitis in cattle worldwide and that the incidence of this disease is steadily increasing [13]. In Poland, cases of infection are also being found with increasing frequency; according to a study by Jagielski et al. [1], *Prototheca* spp. accounted for 8% of mastitis pathogens found and thus ranked third, just after bacteria of *Streptococcus* spp. and *Staphylococcus* spp. Mastitis caused by an infection with algae of *Prototheca* spp. is most often subclinical and is characterized by a marked, steady increase in somatic cell count and a large decrease in milk yield. The treatment for algal mastitis is especially problematic because *Prototheca* spp. do not respond to most currently known treatments, among which are antibiotics and mycotics. The only potential agent demonstrating a therapeutic effect seems to be liposomal amphotericin B (AMB) [10], which is very expensive; therefore, its use in the routine treatment of cattle would not be cost-effective. In addition, *Prototheca* spp. demonstrate an ability to survive over a wide range of temperatures [10,14], making the preventive control of this pathogen much more difficult, which in turn increases the risk of transmission to healthy animals.

All of the above-mentioned factors mean that the only effective and cost-efficient way to stop the spread of protothecal mastitis among animals is to exclude infected animals from the herd [1] or to permanently dry the quarter of the udder.

The most common treatment for mastitis is antibiotic therapy. However, the excessive and prolonged use of antibiotics in animal production—both for treatment and prevention—has greatly contributed to the development of antimicrobial resistance (AMR) among pathogens [15]. This poses a growing threat to both animal and human health, as resistant bacteria can spread through food chains, the environment, and direct contact [16]. In regions like Africa, high volumes of antibiotics are used in livestock, often with limited regulation, resulting in multidrug-resistant strains [15]. Even in Europe and the USA, where stricter policies exist, AMR remains a significant concern due to misuse and inadequate monitoring [17]. Furthermore, antibiotic residues entering the environment can disturb microbial ecosystems. Therefore, it is crucial to develop alternative strategies for mastitis prevention and treatment within a One Health framework [17]. A promising alternative to synthetic agents seems to be extracts and oils of plant origin, which have been known to humankind for a very long time for their health-promoting and antimicrobial properties [18]. Essential oils (EOs) are defined as the volatile, secondary metabolites of plants that give them their characteristic smell or taste. They are produced by more than 17,500 plant species across many angiosperm families, but only about 300 of these are commonly used [19]. EOs are highly complex mixtures of, mainly, terpenes, terpenoids, and phenylpropanoids. They may also contain many other compounds, such as fatty acids, oxides, and sulfur derivatives [18,20]. EOs are increasingly recognized as promising alternatives to antibiotics due to their broad-spectrum antimicrobial properties and generally low toxicity, given at the right dose [21,22,23]. EOs derived from *Thymus vulgaris* (thyme) and *Origanum vulgare* (oregano) show strong antibacterial activity, including against resistant strains, while remaining non-toxic to human cells at effective concentrations [19]. Additionally, when used alongside conventional antibiotics, EOs can enhance antimicrobial efficacy and reduce the required dosage, lowering the risk of side effects and resistance development [19,21,24]. EOs and their individual components have been shown to have biocidal effects against bacteria, yeasts, filamentous fungi, and viruses [18,25,26]. Due to the growing interest in natural products and their wide utilization, it is important to better understand their mode of biological action in order to discover new applications in medicine and agriculture. Some of these products provide an effective and cheaper alternative or addition to synthetic compounds from the chemical industry [27,28].

In summary, it is necessary to continue the search for substances that could potentially help in the control of *Prototheca* spp. infections and that could also be used for treatment and prevention.

The aim of the present study was the in vitro comparison of the antimicrobial properties of AMB and 10 selected, commercially available EOs that could have potential antialgal properties: clove oil (*Syzygium aromaticum*), tea tree oil (*Melaleuca alternifolia*), geranium oil (*Pelargonium graveolens*), lavender oil (*Lavandula officinalis* syn. *L. angustifolia*), bergamot oil (*Citrus bergamia*), rosemary oil (*Rosmarinus officinalis*), cajeput oil (*Melaleuca leucadendra)*, sage oil (*Salvia officinalis* subsp. *lavandulifolia*), thyme oil (*Thymus vulgaris* L.), and oil of oregano (*Origanum vulgare*). The experiment included two strains of *Prototheca bovis* (PRO3 and PRO7) isolated from the quarter milk of cows that had been diagnosed with chronic mastitis.

The analysis was performed by using toxicity test. The obtained results may prove useful for further research into the potential use of substances of natural origin in the control of protothecal infections in cattle. We believe that our research is the first attempt to use methods other than microdilutions, e.g., MIC (minimal inhibitory concentration), MAC (minimal algicidal concentration), or disc diffusion, in studies focusing on the search for new antialgal agents.

## 2. Results

### 2.1. Chemical Compositions of Essential Oils

The results of the analysis carried out on the chemical composition of the tested EOs are presented in Table 1. The most frequently recurring components—at various concentrations—were α-pinene, β-pinene, myrcene, p-cymene, limonene, eucalyptol, linalool, camphor, α-terpineol, β-caryophyllene, and caryophyllene oxide.

The main component of clove oil was eugenol (66.0%). For tea tree oil, the component with the highest concentration was terpien-4-ol (32.1%). Geranium oil had one of the most complex compositions, but it was citronellol that had the highest concentration (30.8%). Linalool (30.9%) and linalool acetate (29.1%) had the highest proportions in lavender oil’s composition. In bergamot oil, limonene was found to be the main constituent (30.6%), while in rosemary oil, components such as α-pinene (20.5%), eucalyptol (24.0%), and camphor (21.9%) predominated. For cajeput oil, eucalyptol accounted for almost half of all components (44.4%). In sage oil, camphor was recorded at the highest concentration (22.1%). Thymol was present at the highest levels in the composition of thyme oil (38.7%), while in the composition of oregano oil, carvacrol accounted for almost half (46.4%).

Monoterpenes formed the main component in the composition of almost all of the oils tested: lavender oil, 92.7%; oregano oil, 94.2%; rosemary oil, 94.9%; sage oil, 98.6%; geranium oil, 95.7%; thyme oil, 95.7%; cajeput oil, 99.0%; and tea tree oil, 93.9%. Monoterpenes accounted for 100% of the components of bergamot oil. The only exception to the very high percentage of monoterpenes was clove oil (0.2%). Sesquiterpenes were the second largest group of compounds making up the chemical oils being tested. Clove oil was the first to stand out with the highest content, 21.6%, which included β-caryophyllene, α-humulene, δ-cadinene, cyclocaryophyllane aldehyde, caryophyllene oxide globulol, and humulene-6,7-epoxide. It was immediately followed by lavender oil (7.1%), tea tree oil (6.8%), oregano oil (5.8%), rosemary oil (5.1%), thyme oil (4.3%), geranium oil (3.2%), sage oil (1.7%), and at the very end, cajeput oil (1.0%).

Only in the composition of clove oil, there were phenylpropanoids, which accounted for as much as 78.2%. Among them, eugenol (66.0%) and eugenol acetate (12.3%) were identified. There were constituents found only in geranium oil that could not be assigned to the groups mentioned above, which distinguished it from the other oils. These constituents accounted for 1.143%. Among these were 6-methyl-5-hepten-2-one, phenylethyl alcohol, and 2-phenylethyl tiglate.

### 2.2. Visual Assessment of Plates

The results of the 96-well plates (n = 8) to which 1% or 2% EO was added, using the XTT assay, are presented in Material and Methods chapter. Each plate was prepared by using the same distribution scheme for the experimental (columns 1–10) and control groups (column 11).

**Figure 1:**
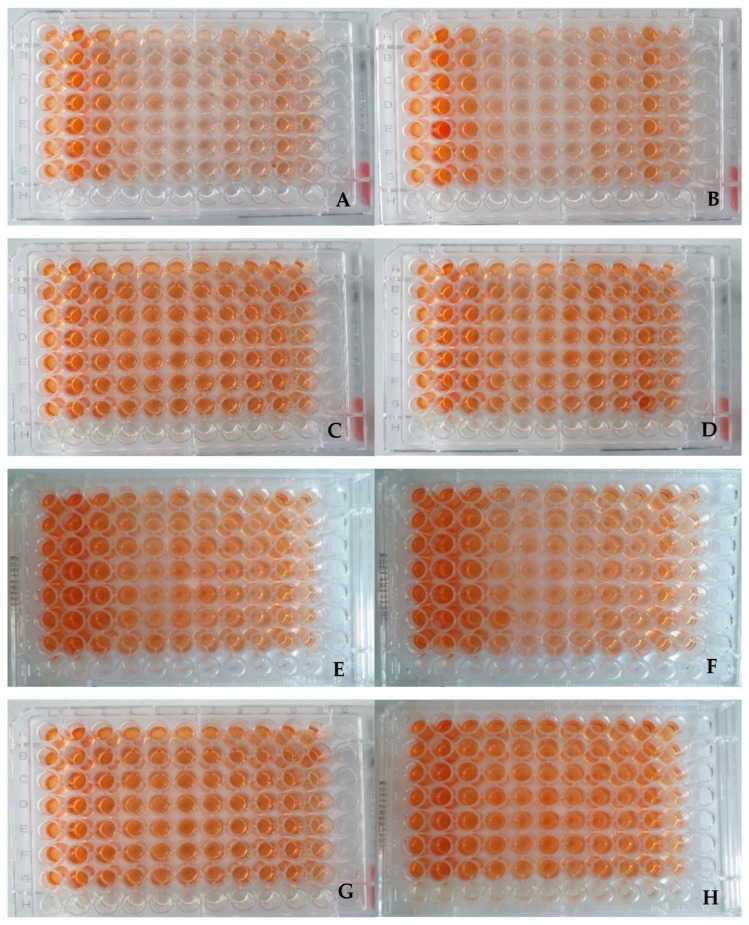
The 96-well plates used in the experiment after 24 h of incubation with XTT reagent. Strain PRO3: (**A**) NaCl, with 1% EO concentration; (**B**) NaCl with 2% EO concentration; (**C**) CampyFood broth medium with 1% oil; (**D**) CampyFood broth medium with 2% oil. Strain PRO7: (**E**) NaCl with 1% oil; (**F**) NaCl with 2% oil; (**G**) CampyFood broth with 1% oil; (**H**) CampyFood broth medium with 2% oil. The plates were prepared according to the following scheme: A1–G1, control samples; 2A–2G, clove oil; 3A–3G, tea tree oil; 4A–4G, geranium oil; 5A–5G, lavender oil; 6A–6G, bergamot oil; 7A–7G, rosemary oil; 8A–8G, cajeput oil; 9A–9G, sage oil; 10A–10G, thyme oil; and 11A–11G, oregano oil.

After the samples had been incubated for 24 h with the XTT reagent, and just before measuring absorbance, a visual evaluation of the plates was performed. The visual evaluation showed that the plates containing 1% oil addition in the medium had a slightly darker color than the samples containing 2% oil (Figure 1). However, a more intense color was observed in the control groups compared with the other samples, especially for the medium containing 0.9% NaCl. Samples containing the CampyFood broth (CFB) medium also had a significantly more intense coloration. During the visual evaluation, no visible differences were observed between the PRO3 (Figure 1A–D) and PRO7 (Figure 1E–H) strains.

### 2.3. Absorbance Measurements

**Figure 2:**
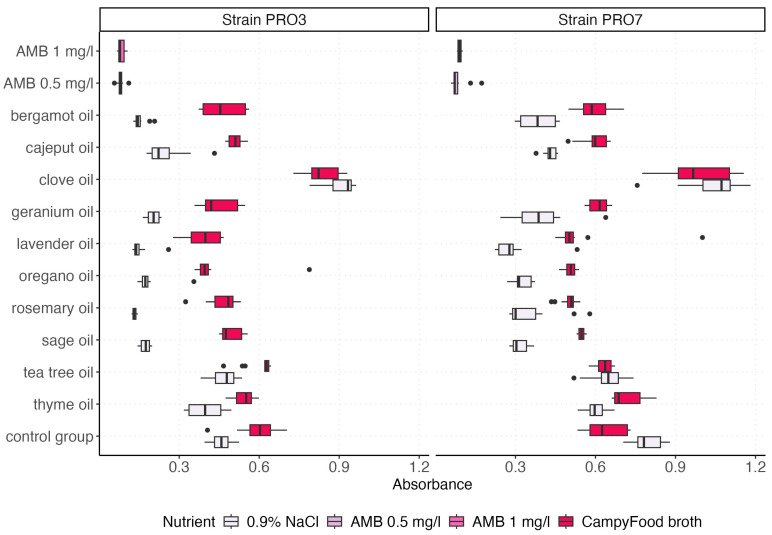
The box plots represent the antimicrobial activity of EOs and control agents (AMB at 0.5 mg/L and AMB at 1 mg/L) in two types of nutrient environments: 0.9% NaCl and CampyFood broth. Lower absorbance values indicate stronger inhibition of bacterial growth. Differences in susceptibility between the two strains (PRO3 vs. PRO7) and nutrient-dependent variability in oil efficacy are clearly observed.

**Figure 3:**
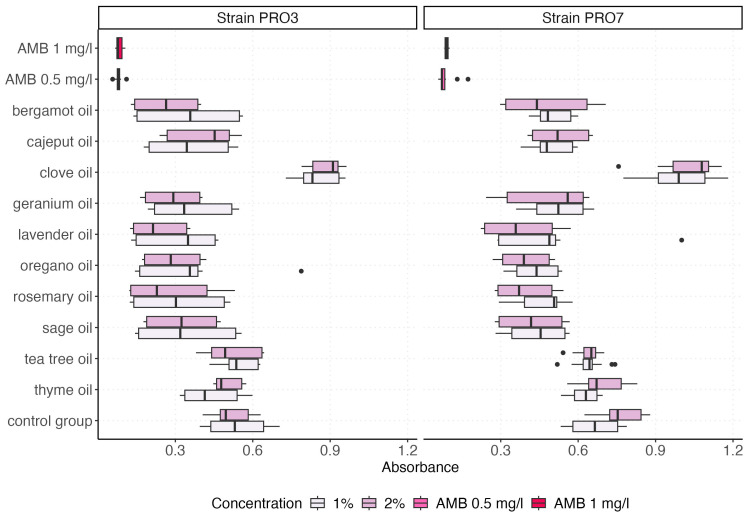
The box plots illustrate the antimicrobial activity of various EOs at two concentrations (1% and 2%), compared with amphotericin B (AMB at 0.5 mg/L and 1 mg/L) and a control group. Absorbance values reflect bacterial growth levels, where lower values indicate stronger inhibition. The differences between the two strains highlight the variable susceptibility of PRO3 and PRO7 to EO treatments.

The visualization of the absorbance value distributions obtained for both *P. bovis* strains (PRO3 and PRO7) across all tested EO and control groups is presented by using box plots, which graphically represent the distribution and variability of the measured parameters (Figure 2 and Figure 3). In Figure 3, we compare the effect of the growth medium (0.9% NaCl vs. CampyFood broth) on the inhibition of both strains by the individual essential oils (at a 1% concentration). In contrast, Figure 3 examines the same set of oils (as well as amphotericin B and the control group) in a single medium but at two concentrations (1% and 2%), allowing us to assess the dose–response relationship. The distribution of absorbance values varied depending on the tested factor (i.e., the type of EO). The highest absorbance values were recorded for clove oil, consistently exceeding those of the control group, regardless of the strain tested. This suggests that clove oil may not exhibit inhibitory effects on *P. bovis* growth under the tested conditions.

In general, the absorbance values differed between the strains, with mean values as follows:

Strain PRO3: 0.396 (95% CI: 0.258, 0.534).

Strain PRO7: 0.531 (95% CI: 0.506, 0.556).

The higher average absorbance for strain PRO7 compared with PRO3 suggests that strain PRO7 may be less susceptible to the biocidal activity of the tested EOs. This difference was further confirmed by a Mann–Whitney U test with continuity correction, which demonstrated a highly significant difference in absorbance distributions between the two strains (*W* = 36,316, *p* = 2.846 × 10^−14^). The test supports the alternative hypothesis that the location shift between the two strains is statistically significant, further indicating strain-dependent variability in susceptibility to antimicrobial treatments.

Furthermore, several EO—such as oregano, thyme, and tea tree oils—demonstrated a marked inhibitory effect on both strains, reflected in significantly reduced absorbance values, in some cases comparable to those obtained with AMB, which was used as a reference antifungal agent.

A detailed summary of the descriptive statistics for the absorbance values across all treatment groups is presented in Table 2. The results show marked differences in antimicrobial activity depending on the agent (AMB or EO) and its concentration.

Among all tested EOs, clove oil exhibited the highest mean absorbance values, with 0.93 at 1% and 0.96 at 2%, suggesting limited or no antimicrobial efficacy under the tested conditions. In contrast, AMB demonstrated the strongest antibacterial activity, with the lowest mean absorbance values of 0.08 (AMB 0.5 mg/L) and 0.09 (AMB 1 mg/L), accompanied by the lowest standard deviations (SD = 0.02 and 0.01, respectively), indicating both potency and consistency.

Tea tree oil and thyme oil displayed intermediate levels of antimicrobial activity. Tea tree oil yielded mean absorbance values of 0.59 (1%) and 0.58 (2%), while thyme oil resulted in values of 0.53 (1%) and 0.60 (2%). These findings suggest a moderate biocidal effect with relatively low variability (SD = 0.08–0.13). Similarly, oregano oil showed promising activity with mean absorbance values of 0.38 (1%) and 0.34 (2%), coupled with modest standard deviations (SD = 0.15 and 0.12).

Lavender, rosemary, and sage oils also demonstrated moderate inhibitory effects, with mean absorbance values ranging from 0.30 to 0.39 across concentrations. Of note, lavender oil at 1% showed higher variability (SD = 0.19), whereas rosemary oil and sage oil maintained relatively consistent outcomes.

Interestingly, bergamot and cajeput oils showed differing dose–response trends. For bergamot oil, the mean absorbance decreased from 0.43 (1%) to 0.37 (2%), while cajeput oil displayed an opposite trend, with a higher absorbance at 2% (mean = 0.47) compared with 1% (mean = 0.43). A non-linear or oil-specific concentration effect may explain these observations. Geranium oil also showed a concentration-dependent effect, with improved antimicrobial activity at 2% (mean absorbance 0.38) compared with 1% (mean = 0.44).

The control group demonstrated consistently high absorbance values at both 1% and 2% (mean = 0.60 and 0.64, respectively), reflecting uninhibited bacterial proliferation. Across all groups, the standard error of the mean (SE) remained low (typically ≤ 0.03), ensuring robust estimation of central tendency despite differences in dispersion.

### 2.4. Group Comparison

To assess the impact of different essential oils on the growth of *P. bovis*, a non-parametric Kruskal–Wallis rank sum test was performed separately for strains PRO3 and PRO7. The results indicated highly significant differences in absorbance values among treatment groups for both strains. For the strain PRO3, the test yielded a chi-squared statistic of 199.78 (df = 12, *p* < 2.2 × 10^−16^), while for the strain PRO7, the test statistic was even higher (χ^2^ = 239.16, df = 12, *p* < 2.2 × 10^−16^). These results statistically confirm that the type of essential oil significantly influences bacterial growth inhibition, justifying further pairwise comparisons.

**Figure 4:**
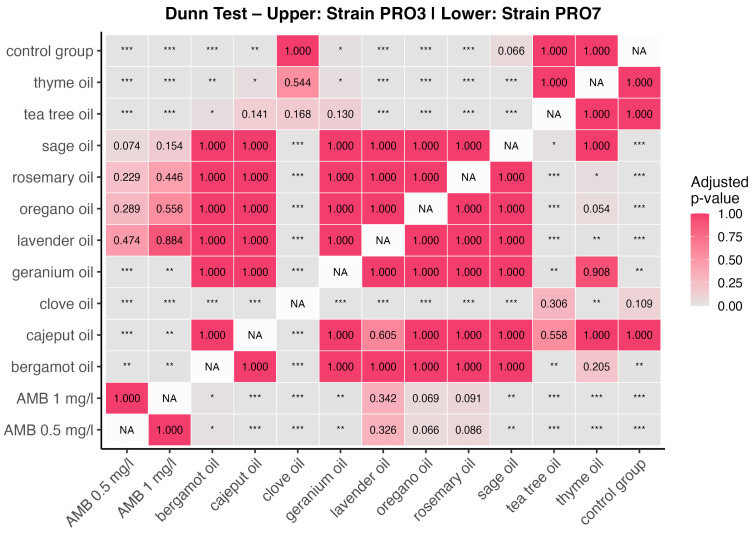
Heatmap of adjusted *p*-values from Dunn’s post hoc test comparing absorbance values across *EO* treatments in PRO3 and PRO7 strains. The upper triangle represents pairwise comparisons for the strain PRO3, while the lower triangle corresponds to the strain PRO7. The color gradient indicates the adjusted *p*-values, with lighter shades representing stronger statistical significance. Asterisks denote the significance thresholds (* for *p* < 0.05, ** *p* < 0.01, and *** *p* < 0.001). NA indicates self-comparisons.

To identify specific group differences, Dunn’s post hoc test with multiple comparison correction was applied. The results are presented in Figure 4 as a heatmap of adjusted *p*-values, with the upper triangle corresponding to PRO3 and the lower triangle to PRO7. Each cell represents a pairwise comparison between two treatments, where color intensity reflects statistical significance (darker = lower *p*-value). Additionally, asterisks (*) indicate the significance thresholds (*p* < 0.05, *p* < 0.01, and *p* < 0.001).

For the strain PRO3, Dunn’s test revealed numerous significant differences. Notably, thyme oil, tea tree oil, clove oil, and geranium oil showed consistent and highly significant differences (*p* < 0.001) when compared with the control group and several other EOs. Clove oil differed significantly from nearly all other treatments, which aligns with previous observations of its distinctively high absorbance values, likely indicating growth-promoting effects rather than antimicrobial activity.

In contrast, AMB at both 0.5 mg/L and 1 mg/L exhibited significantly lower absorbance values than most essential oils, reaffirming its role as a positive control with strong antimicrobial activity. AMB reduced algal viability by an average of 88% compared with the control groups. Pairwise comparisons involving AMB showed statistical separation from nearly all EOs, except in some cases where oils such as oregano or tea tree oil approached similar inhibitory effects.

For the strain PRO7, although the general pattern of significance was similar, fewer comparisons reached statistical significance, indicating a lower overall sensitivity to treatment. Nevertheless, oils such as sage, rosemary, and thyme remained statistically distinguishable from control or weaker treatments (e.g., bergamot and clove), especially in comparisons with AMB or the control group. This observation is consistent with previously noted differences in baseline absorbance between strains, where PRO7 exhibited higher overall absorbance values and reduced variability in the response.

The combined interpretation of the Kruskal–Wallis test and Dunn’s post hoc analysis clearly supports the conclusion that essential oils differ significantly in their antimicrobial activity and that this activity is strain-dependent, with PRO3 demonstrating a higher degree of statistical separability among treatments.

The analysis reveals statistically significant differences among several EOs, particularly between clove oil and other treatments, as well as between the control group and high-efficacy oils (e.g., thyme, tea tree, and oregano). AMB consistently differed from most oils, confirming its superior antialgal activity. The pattern of significance was more pronounced in the strain PRO3, indicating a higher sensitivity to treatment compared with the strain PRO7.

### 2.5. Interactions

**Figure 5:**
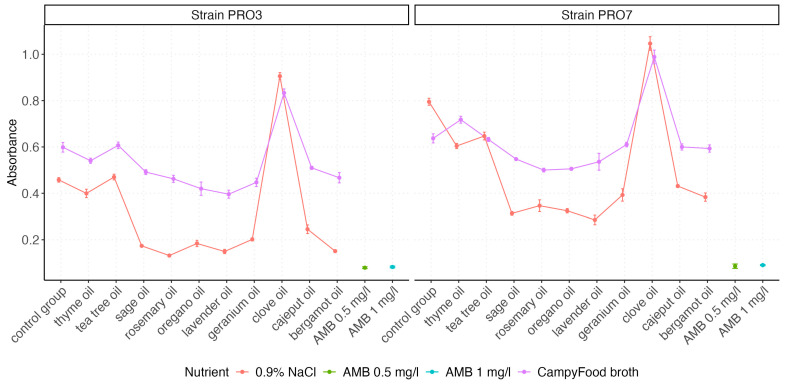
Line plots illustrate mean absorbance values (±SE) by oil and nutrient environment. Lower absorbance values indicate stronger inhibition.

Further analyses explored interaction effects. Figure 5 illustrates the interaction between the EO type and the nutrient environment. CampyFood broth consistently yielded higher absorbance values, suggesting reduced antimicrobial efficacy under nutrient-rich conditions. In contrast, oils like sage, oregano, rosemary, and lavender showed stronger inhibitory activity in 0.9% NaCl, particularly in the strain PRO3.

**Figure 6:**
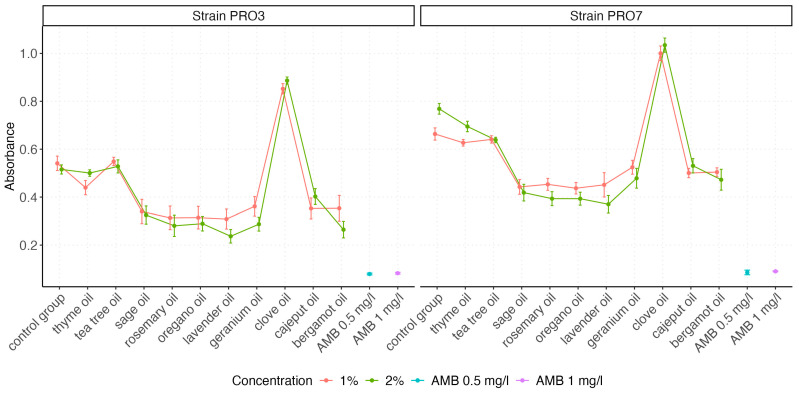
Line plots show mean absorbance (±SE) for each oil at 1% and 2% concentrations. Lower absorbance indicates stronger antimicrobial activity.

Figure 6 presents the interaction between the oil type and the concentration. In both strains, certain oils (oregano, sage, lavender, and rosemary) demonstrated slightly better efficacy at 2%. In contrast, some oils, such as thyme and tea tree, showed minimal differences, suggesting a concentration-independent plateau effect.

### 2.6. Algal Viability

**Figure 7:**
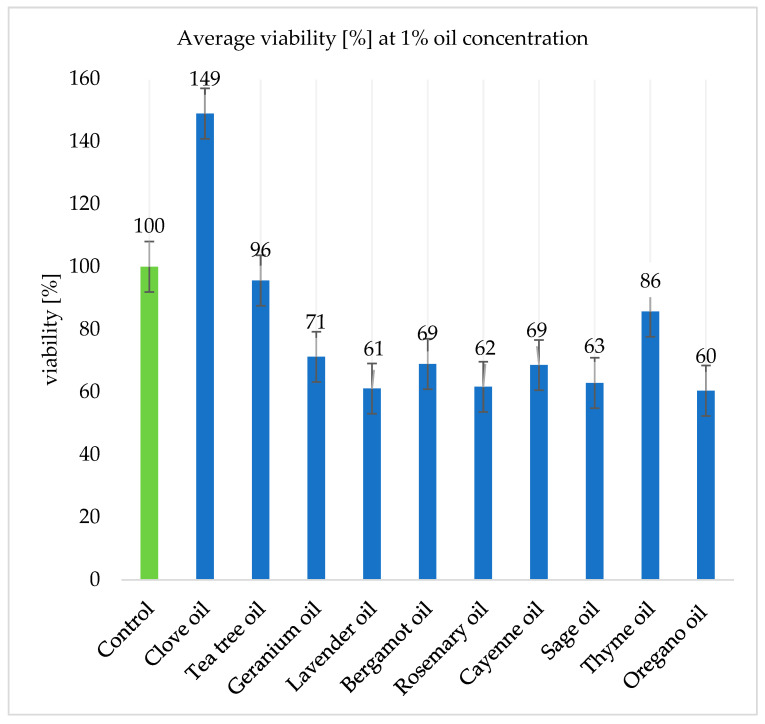
The average viability of the tested *Prototheca bovis* strains with essential oils at a concentration of 1%.

**Figure 8:**
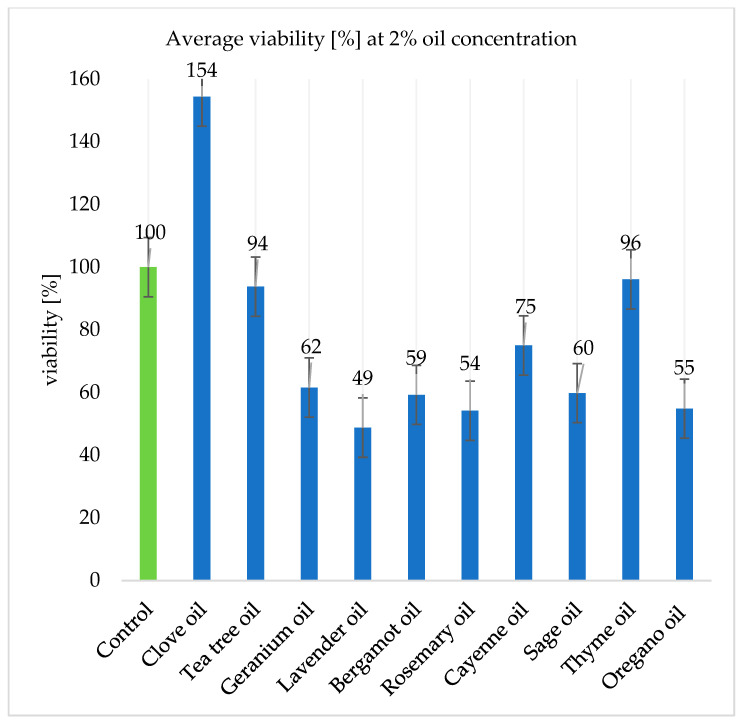
The average viability of the tested *Prototheca bovis* strains with essential oils at a concentration of 2%.

The results presented in Figure 7 and Figure 8 present the algal survival rates. The viability values for samples containing 1% oils were on average higher than those for samples containing 2% oils. The viability values were calculated in reference to the control group, whose value is presented as 100%.

Clove oil at a concentration of 2% increased the survival of *Prototheca* spp. cells by as much as 54.39% on average. However, this could have been caused by a number of factors: an unexpected colorimetric reaction between one of the oil components and the XTT reagent, errors during laboratory work (pipetting), or errors related to the preparation of suspensions containing the pathogen.

The tea tree and thyme oils reduced viability rates by, on average, 6.23% and 14.09%, respectively. However, at a concentration of 2%, thyme oil had a lower biocidal effect, reducing pathogens viability by only 3.94%. Geranium oil reduced survival rates by an average of 38.44%. Bergamot and cajeput oils reduced cell survival by 40.76% and 25.00%, respectively.

At a concentration of 2%, lavender, rosemary, and oregano oils showed the highest antimicrobial effect, reducing the survival of both of the *Prototheca bovis* strains tested by 51.21%, 45.83%, and 45.15%, respectively (Figure 8).

## 3. Discussion

### 3.1. EO Characteristics

The analysis of the chemical composition of the oils confirmed the literature reports. As much as 66.0% eugenol was found in clove oil—this is the component that is usually found in the highest amount [31,32]. In tea tree oil, the main component is usually terpinene-4-ol, accounting for about 40% of the oil [33,34], and in the current case, it was found at a concentration of 32.1%. The essential oil extracted from rose geranium contains mainly citronellol, citronellyl acetate, citronellyl formate, and geraniol [35,36]. According to this study, citronellol had the highest concentration (30.8%), followed by geraniol (15.6%). The main constituents of lavender oil are usually linalyl acetate, linalool, p-cymene, 1,8-cineole, terpinen-4-ol, and camphor [25]; our study confirmed this, as linalool had the highest concentration (30.9%), immediately followed by linalool acetate (29.1%). The main components of bergamot oil are usually limonene, linalool, and linalyl acetate [37]. The highest concentration among all components is that of limonene; according to our study, the oil contained 30.6% limonene. Rosemary essential oil is mainly composed of monoterpenes such as 1,8-cineole (eucalyptol), camphor, and α-pinene [38]. The oil tested in this study contained primarily α-pinene (20.5%), eucalyptol (24.0%), and camphor (21.9%). In the case of cajeput oil, one of the main chemical constituents is eucalyptol, whose content ranges from 15 to 60% [39]. The oil analyzed here contained 44.4% of this component. The main components of sage oil are usually borneol, camphor, 1,8-cyneol, camphene, limonene, α-pinene, β-pinene, α-thujone, β-thujone, α-humulene, sesquiterpene derivatives, and β-caryophyllene [40]. In the oil studied here, the majority of the composition was represented by camphor (22.1%) and, again, eucalyptol (10.4%). As reported in a study by De Martino et al. [41], among others, the main constituents of thyme oil are thymol (36–55%) and p-cymene (15–28%); this was also confirmed by our study, where thymol accounted for 38.7%, immediately followed by p-cymene (20.5%). The last oil tested, oregano oil, contained as much as 46.4% carvacrol. According to the literature, the main terpenes identified in different oregano species are usually carvacrol, thymol, γ-terpinene, and p-cymene [42].

### 3.2. Prototheca Genus Taxonomy

The taxonomy of the genus *Prototheca* has already been revised in the past, and currently, the phenotypic description is not precise enough for algal identification. Nowadays, molecular analysis, for example, based on the *cytb* gene, could be used in modern research [43]. One of the major developments, which is especially important for researchers and breeders connected with the dairy industry, is a new change in the taxonomy for species inducing mastitis in cattle. For example, *P. ciferrii* and *P. bovis* were previously classified as *P. zopfii* gen. 1 and gen. 2, respectively. In the case of *P. blaschkeae*, the species is now also classified as *P. ciferrii*. These changes in the taxonomy of microalgae mean that it may become more difficult to analyze the results of studies and the complexities affecting their correct identification.

### 3.3. Current Methods of Protothecosis Treatment Using EO and AMB

The number of references focusing on the biocidal effects of EOs against *Prototheca* spp. that have been isolated from cows with mastitis are still limited. This paper is probably the first one using methods other than MIC, MAC, or disc diffusion in studies focusing on the search for new antialgal agents.

In a study by Grzesiak et al. [44], cinnamon, geranium, clove, thyme, lavender, basil, rosemary, and clary sage EOs inhibited the activity of *P. zopfii* isolates, with MIC (minimum inhibitory concentration) values ranging from 0.2 to 10.5 μL/mL. Cinnamon, clove, and thyme demonstrated the highest activity against the tested *P. zopfii* strains at concentrations ranging from 0.6 to 1.0 μL/mL. The authors proved that the examined strains were resistant to most of the recommended antifungals but sensitive to nystatin. However, in further research, the same authors presented a study in which one *P. zopfii* strain was resistant to nystatin (100 µg) [45]. In contrast, Nardoni et al. [46] revealed that *Prototheca zopfii* and *Prototheca blaschkeae* strains were susceptible to conventionally used antifungal drugs such as amphotericin B and posaconazole but were resistant to voriconazole (MIC = 6 μg/mL). The authors pointed out that the lowest MICs (0.75%) were obtained for *Citrus paradisi* for both algal species; yet, *P. zopfii* appeared to be more sensitive to EOs in comparison with *P. blaschkeae*.

The differences in the EO activity can also be observed when considering different strains (field or referent). In our study, both strains were found to be sensitive to AMB, which reduced their viability by an average of 88% compared with the control groups. Interestingly, the strain PRO7 was less sensitive to this antibiotic. Although this antifungal drug exhibits satisfactory antimicrobial activity, its use in the treatment of *Prototheca* spp. infections in cattle is expensive and may be associated with adverse effects, which further limits its application in veterinary practice. Research conducted by Catoi et al. [47] with the *P. zopfii* strain isolated from cows and a *Prototheca wickerhamii* referent strain, demonstrated the highest cytotoxic effect in the case of savory (*Satureja hortensis*), mint (*Mentha piperita*), and tea tree (*Melaleuca alternifolia*) oils. In general, the authors proved that *P. wickerhamii* was more sensitive to all natural products. Depending on the region of the world, various local plants or herbs could be a source of EO, which would ensure a lack of problems connected with importing plants, seeds, or oils of different qualities. The antialgal activity of extracts from the Iranian plants *Allium cepa, Apium graveolens*, *Ficus carica*, and *Actinidia deliciosa* and of *Ferula galbaniflua boiss. et buhseessential* oil was recently evaluated by the Abbasi Sani et al. [48]. The results showed that the extract from *A. deliciosa* had the lowest pH and probably, because of this, revealed the highest biocidal effect.

A different approach could focus only on the phytochemicals present in EOs. Nojo et al. [49] tested carvacrol, citral, and thymol as biocidal agents against *Prototheca* strains isolated from animals. The MIC ranges of these phytochemicals, for all isolates, varied from 0.03% to 0.125% for carvacrol, 0.03% to 0.25% for citral, and 0.06% to 0.25% for thymol. According to these results, these EO constituents may be potential agents in future research and may be implemented as components of commercial products used in mastitis prevention or protothecosis treatment. These findings are consistent with the results presented in this manuscript, suggesting that oregano oil had the highest carvacrol level and also included thymoml. However, further in vivo studies are necessary in order to verify their influence on udder cells, cow welfare, and milk quality.

Almost all tested EOs, with the exception of clove oil, reduced microalgal survival by at least 3.94% (thyme oil). Interestingly, clove oil differed the most in composition from the other oils tested—it was the only one to contain the fewest monoterpenes, including eucalyptol (0.2%). Three plant oils—lavender, rosemary, and oregano—showed clear inhibitory effects on the proliferation of microalgal cells of *Prototheca* spp. Lavender oil at a concentration of 2% showed the strongest biocidal effect against *Prototheca* spp., reducing microalgal survival by 51.21%.

Thorough studies on the biocidal properties of EO against *Prototheca* spp. using a toxicity test with XTT reagent have not yet been carried out, but the biocidal properties of EOs against fungi, bacteria, and even viruses have previously been well documented.

Lavender oil has been found to have biological activity against many strains of bacteria and fungi. It has also been suggested that it may be useful in the treatment of antibiotic-resistant bacterial infections. For example, oil extracted from the species *L. angustifolia* shows in vitro activity against MRSA (methicillin-resistant *Staphylococcus aureus*) and VRE (vancomycin-resistant *Enterococcus faecalis*) at concentrations of less than 1% [50]. Lavender oil has also shown antimicrobial activity against piperacillin-resistant *Escherichia coli* strain J53 R1. The activity of lavender oil was also investigated for 24 *Listeria monocytogenes* strains, where the minimum inhibitory concentration (MIC) was 2.5–5.0 μL/mL. In the case of chloramphenicol-resistant *Listeria monocytogenes* L120, the MIC value was 1.3 μL/mL [51]. Rosemary oil reduced the survival rate of the tested *Prototheca bovis* strains by 45%. The oil has previously been shown to have antimicrobial potential against many microorganisms, most potently against *Candida albicans*, *Escherichia coli*, *Salmonella typhi*, *Salmonella enteritidis*, and *Shigella sonnei*.

The EO effect has also even been proven against drug-resistant strains [52,53]. In the current study, oil of oregano reduced the survival rate of microalgae by 45.15%. Numerous studies, both in vitro and in vivo, prove the bactericidal and antifungal effects of oregano oil [54,55]. Among the microorganisms tested for sensitivity to the oil by Adame-Gallegos et al., Gram-negative bacterial strains such as *Salmonella typhimurium*, *Escherichia coli*, *Klebsiella pneumoniae*, *Yersinia enterocolitica*, and *Enterobacter cloacae*, as well as Gram-positive strains such as *Staphylococcus epidermidis*, *Listeria monocytogenes*, and *Bacillus subtilis*, were included. Fungi, on the other hand, included species such as *Aspergillus flavus*, *Aspergillus ochraceus*, *Aspergillus niger*, *Candida albicans*, *Candida glabrata*, and *Trichophyton mentagrophytes* [54]. Interestingly, according to a study by Massa and colleagues [56], oregano essential oil showed the most potent biocidal properties against *Candida glabrata* out of all the oils tested at that time. The three oils that produced the best results differed in their composition. This may suggest that the individual components have different biological activity against *Prototheca* spp. The components that distinguished these essential oils from the others in terms of their concentration were linalool, linalyl acetate, alpha-pinene, and carvacrol. These compounds were also present in other tested EOs but at significantly lower concentrations. For example, bergamot oil contained 18.7% linalool, which is 12.2% less than that in lavender oil. It is possible that these specific compounds are responsible for the biocidal activity against the tested microalgal strains. Interestingly, lavender oil had the most complex composition of all these three EOs. It is also worth noting that this oil contained components that were not present in the composition of the other oils tested. Ingredients such as plinol A (0.1%), trans-2-pinanol (0.2%), 3-pinalol (0.3%), plinol (0.7%), 1-terpineol (0.1%), trans-β-terpineol (0.2%), monoterpenoid acetate (0.2%), plinol D (0.1%), and lavandulyl acetate (0.4%) were identified. However, similarities were discovered in the three oils in the form of components such as α-pinene, myrcene, p-cymene, eucalyptol, linalool, camphor, and β-caryophyllene. On the other hand, EOs that contained the same components, even at higher concentrations, did not satisfactorily reduce microalgal viability. For example, cajeput oil contained twice as much eucalyptol (44.4%) as rosemary oil (24%); sage oil contained 22.1% camphor, while lavender oil contained 21.9%; thyme oil had as much as 38.7% thymol, while oregano oil had only 17.5% of this compound. This may be due to the fact that the individual oil components or even their specific concentrations may work better in synergy with the other components in a particular EO.

In conclusion, EOs and their components have well-known antimicrobial properties and are used in several fields of human and veterinary medicine. For this reason, continuing research in the context of protothecosis or mastitis caused by microalgae seems to be the right direction, especially due to their resistance to commonly used antifungal drugs.

## 4. Materials and Methods

### 4.1. Biological Material

Quarter milk samples were collected in November 2023 at two dairy cattle farms in the Kuyavian-Pomeranian and West Pomeranian voivodeships, which maintained 450 and 360 dairy cows of the Polish Holstein-Friesian breed, respectively. For the experiment, cows were selected on the basis of milk performance reports conducted by the Polish Federation of Cattle Breeders and Milk Producers for the breeders. Quarter milk samples (n = 17) were taken from cows with chronic subclinical mastitis, with an SCC (somatic cell count) > 1,000,000/mL, occurring for at least three months, and a positive California Mastitis Test (CMT) with a score of +++ [57]. The obtained material was frozen at −20 °C and transported to the Institute of Animal Sciences at Warsaw University of Life Sciences for further analysis.

### 4.2. Tested Essential Oils

Commercially available EOs (Naturalne aromaty, Bochnia Poland) were used: clove oil (*Syzygium aromaticum*), tea tree oil (*Melaleuca alternifolia*), geranium oil (*Pelargonium graveolens*), lavender oil (*Lavandula officinalis* syn. *L. angustifolia*), bergamot oil (*Citrus bergamia*), rosemary oil (*Rosmarinus officinalis*), cajeput oil (*Melaleuca leucadendra*), sage oil (*Salvia officinalis* subsp. *lavandulifolia*), thyme oil (*Thymus vulgaris* L.), and oregano oil (*Origanum vulgare*). A number was randomly assigned to the control group and to each EO to code the data obtained from the toxicity tests for statistical analysis (Table 3.).

### 4.3. Analysis of Chemical Composition

The chemical composition of the EOs was analyzed by using an Agilent 7890A gas chromatograph with an Agilent 5975C mass spectrometer (Agilent Technologies, Santa Clara, CA, USA).

The single essential oil (5 μL) was dissolved in 395 μL of pyridine; then, 1 μL of this sample was injected into the GC/MS apparatus. The injector worked in split (1:10) mode at a temperature of 280 °C. Chromatographic separation was performed in a fused silica column HP-5MS (30 m × 0.25 mm × 0.25 μm) at a helium flow rate of 1 mL/min. The initial column temperature was 50 °C and was raised at a rate of 3 °C/min up to 260 °C. The ion source and quadrupole temperatures were 230 °C and 150 °C, respectively. The mass spectrometer applied an ionization energy of 70 eV. A range of 29–600 units in full scan mode was used for detection. After integrating the peaks on the chromatogram, the percentage content of the compounds within the total ion current (% TIC) was calculated. Mass spectrometry and the retention index were used to identify the compounds. The NIST 2020 [58], Wiley Registry of Mass Spectral Data 2020 [59], Tkachev [30], and Adams [29] mass spectral libraries were used for mass spectrometric analysis [29,30,47,58]. The retention times of the C8–C40 n-alkanes were used for the calculation of the experimental retention indices of the compounds.

### 4.4. Isolation of Prototheca Species

**Figure 9:**
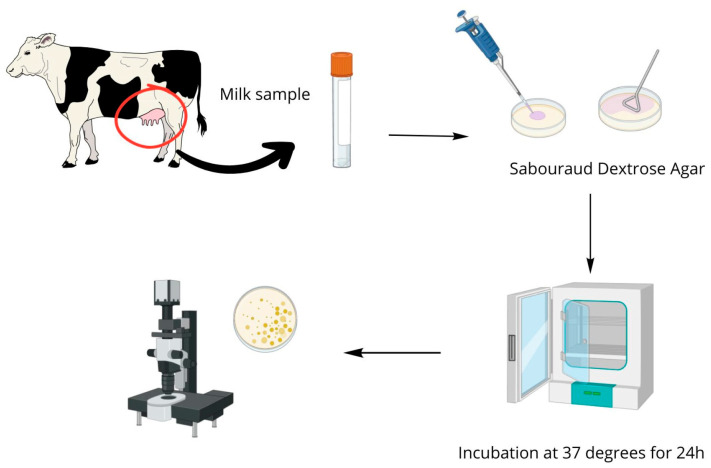
A simplified diagram showing the procedure for isolating the microalgae from the quarter milk samples.

The biological material (quarter milk samples) was transported to a laboratory of the Institute of Animal Science at Warsaw University of Life Sciences and subjected to standard microbiological procedures by using differential selection media for the isolation of *Prototheca* spp.: Sabouraud Dextrose Agar (Oxoid, ThermoFisher Scientific, Horsham, UK) and Prototheca Isolation Medium [60]. The cultures were made directly from the biological material by applying them to the surface of Petri dishes (100 μL) and then left to incubate under aerobic conditions at 37 °C for 24 h to isolate the microorganisms. The preliminary identification of microalgae of *Prototheca* spp. was based on the colony morphology of the cultured pathogens and microscopic analysis after lactophenol blue staining. Two isolated microorganisms, initially identified as strains of *Prototheca* spp., were used for the experiments as follows: PRO3 and PRO7. A simplified scheme showing the procedure of pathogen isolation is shown in Figure 9.

### 4.5. Genetic Analysis

The identification of the isolated *Prototheca* species was based on differences in *cytb* gene sequencing, according to methods presented by Jagielski et al. [43,61,62].

The extraction of DNA material was performed by using the GeneMATRIX Environmental DNA & RNA Purification Kit (EURx, Gdansk, Poland). The extraction process was carried out according to the manufacturer’s instructions. A spectrophotometer (NanoDrop ND-1000, Thermo Fisher Scientific, Waltham, USA) was used for DNA level quantification. The material was stored at 4 °C.

PCR amplification and sequencing were performed by using reaction mixtures with a total volume of 30 μL. Each sample contained 10 ng of DNA (1 μL), 18 μL of Color Taq PCR MasterMix (EURx, Gdansk, Poland), 1 μL of each of the following primers (0.2 μM each): *cytb*-F1 (5’-GyGTwGAACAyATTATGAGAG-3’) and *cytb*-R1 (5’-wACCCATAArAArTACCATTCWGG-3’).

The PCR conditions in the thermocycler were as follows: 3 min at 95 °C, followed by 35 cycles of 30 s at 95 °C, 30 s at 50 °C, and 30 s at 72 °C, and a final extension at 72 °C for 5 min.

Agarose gel (1%) was used in electrophoresis to visualize the amplified products that had been stained with ethidium bromide (5 mg/L) and then exposed to UV light. The obtained amplicons were purified by using Short DNA Clean-Up (EURx, Gdansk, Poland). In the next step, the amplicons were sequenced by using the same primers as those used for the amplification process.

The investigated sequences revealed the isolation of two *Prototheca bovis* strains. The obtained sequences were deposited in Clone Professional Manager Suite 8. The assembled sequences were compared with the references available in the Prototheca-ID web application [63] and the NCBI GenBank database. The strains received positive verification, so their unique sequences formed the basis for the GenBank number (Table 4).

### 4.6. Toxicity Test

The strains PRO3 and PRO7 were prepared for analysis as a colony suspension with an optical density of 1.0 on the McFarland scale, in 0.9% NaCl and in CampyFood broth liquid medium (BioMérieux, Marcy-l’Étoile, France) to assess if the medium utilized affects the viability of *Prototheca bovis* cells.

**Figure 10:**
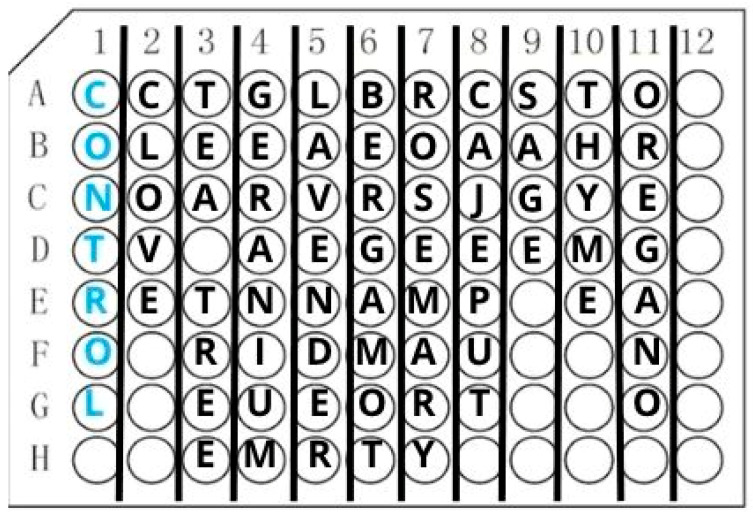
Diagram according to which samples were transferred to 96-well plates. Plate scheme: A1–G1, control samples; 2A–2G, clove oil; 3A–3G, tea tree oil; 4A–4G, geranium oil; 5A–5G, lavender oil; 6A–6G, bergamot oil; 7A–7G, rosemary oil; 8A–8G, cajeput oil; 9A–9G, sage oil; 10A–10G, thyme oil; and 11A–11G, oregano oil.

Samples were prepared in 2 mL Eppendorf tubes containing 1% (*v*/*v*) or 2% (*v*/*v*) of each tested EO. The negative controls contained no addition of EOs, and in the positive controls, AMB at concentrations of 0.5 mg/L and 1 mg/L was used. Further, 5% DMSO (dimethylsulfoxide) was used as the solvent for the oils and AMB. The volume of each prepared samples was 1 mL, and a total of 88 samples were prepared for analysis. The prepared samples were incubated for 24 h on a vortex at 37 °C under aerobic conditions. After the incubation, the samples were transferred to standard polystyrene 96-well plates in seven replicates of 50 µL each, according to the scheme shown in Figure 10.

The evaluation of the algicidal properties of the EOs was performed by using the cytotoxicity test. The Cell Proliferation Kit II assay (XTT) (Merck, Darmstadt, Germany) is used to measure cellular metabolic activity as an indicator of cell viability, proliferation, and cytotoxicity. This colorimetric assay is based on the reduction of a yellow tetrazolium salt (sodium salt of 2,3-bis [2-methoxy-4-nitro-5-sulfophenyl]-2H-tetrazolium-5-carboxanilide) to an orange formazan dye by metabolically active cells. This test is commonly used in studies of cell proliferation in response to various toxicity agents but has also been used in studies on microorganisms [64,65,66].

**Figure 11:**
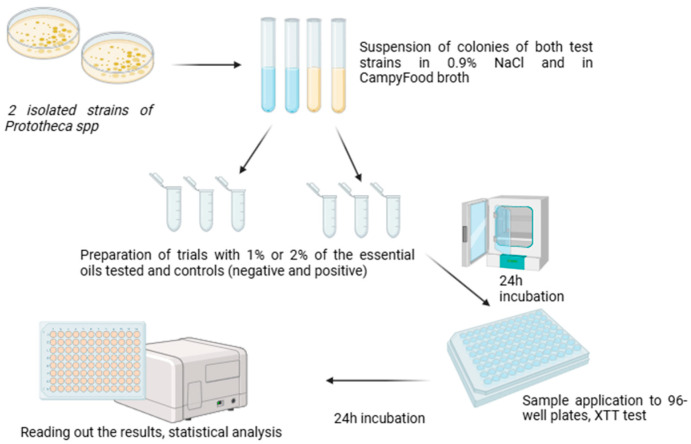
Scheme of sample preparation for toxicity test.

The XTT assay was performed according to the manufacturer’s instruction: A volume of 20 µL of XXT reagent was added to each well after the 24 h incubation. Then, the 96-well plates were secured with parafilm and re-incubated also on a vortex for 24 h at 37 °C under aerobic conditions. After the required 24 h, the absorbance was measured for each sample by using an Infinite200 PRO^®^ plate reader (Tecan Trading AG, Männedorf, Switzerland) at 450/690 nm. A simplified diagram showing sample preparation for the toxicity test is shown in Figure 11.

### 4.7. Statistical Analysis

All statistical analyses were performed by using the R statistical computing environment (version 4.2.3; R Core Team, 2023) on macOS 15.3.2. Data preprocessing, statistical testing, and graphical visualization were conducted in accordance with current standards of reproducible data analysis workflows [67,68,69]. To assess differences in absorbance values between the *P. bovis* strains (PRO3 and PRO7), a Wilcoxon rank sum test (Mann–Whitney U test with continuity correction) was applied. This non-parametric test, which does not assume normal distribution, was used to evaluate whether the distribution of absorbance values differed significantly between the two strains. To examine differences among various EOs and the control treatments, a Kruskal–Wallis rank sum test was performed separately for each strain. Post hoc pairwise comparisons were subsequently conducted by using Dunn’s test with *p*-value adjustment for multiple testing. The graphical representations of the results, including box plots, line plots, and heatmaps of adjusted *p*-values, were created by using the ggplot2 package (version 3.5.2). Descriptive statistics and summary measures, such as mean, standard deviation, and standard error of the mean, were calculated by using functions from the tidyverse ecosystem. The entire statistical workflow was supported by the following R packages: report (version 0.6.1), FSA (version 0.10.0), ggplot2, readxl (version 1.4.5), dplyr (version 1.1.4), tidyr (version 1.3.1), and colorspace (version 2.1.1). Package citations and reporting were generated by using the report package to ensure the transparency and reproducibility of the analyses [70,71,72,73,74,75,76,77].

## 5. Conclusions

The three EOs of lavender, rosemary, and oregano demonstrated a significant inhibitory effect on the proliferation of cells of microalgae of *Prototheca bovis* strains at concentrations of 1% and 2%. However, the strongest biocidal effect against *Prototheca bovis* strains was evidenced by lavender oil at a concentration of 2%, which reduced pathogen viability to 49% compared with the control group. Despite the fact that AMB reduces the viability of microalgae by almost 90%, its practical use in vivo is still limited by the high cost of treatment or its numerous side effects. Further research into the practical use of lavender, rosemary, and oregano EOs may contribute to more effective control or prevention methods for mastitis caused by *Prototheca* spp. This paper is probably the first one using toxicity tests as a research method in studies focusing on development for new antialgal therapies. However, further research is needed, particularly into the properties of the individual components of essential oils, as well as the safety of their use, for example, by evaluating their effects on healthy cells. More extensive in vivo studies are also needed in the future to assess the potential of using essential oils in animal models.

## Figures and Tables

**Table 1:** The composition of the tested essential oils.

Compound	RIexp.	RIlit.	Clove	Tea Tree	Geranium	Lavender	Bergamot	Rosemary	Cajeput	Sage	Thyme	Oregano
			TIC (%)	TIC (%)	TIC (%)	TIC (%)	TIC (%)	TIC (%)	TIC (%)	TIC (%)	TIC (%)	TIC (%)
Monoterpenes:			0.2	93.2	95.7	92.9	100	94.9	99.0	98.3	95.7	94.2
tricyclene	924	921 a	-	-	-	-	-	-	-	0.2	-	-
3-thujene	929	926 b	-	-	-	-	0.3	0.1	1.3	-	-	-
α-pinene	934	932 a	-	3.7	0.9	0.2	1.6	20.5	5.2	8.2	2.3	2.8
camphene	949	946 a	-	-	-	-	-	1.8	-	3.8	-	-
sabinene	974	973 b	-	0.3	-	-	1.9	-	-	0.6	-	-
β-pinene	977	974 a	-	0.3	0.7	-	9.2	12.3	2.0	2.3	2.1	2.2
p-menth-3-ene	984	984 a	-	0.2	-	-	-	-	-	-	-	-
myrcene	991	988 a	-	-	-	0.3	1.3	0.5	1.1	0.6	1.3	0.3
p-mentha-1(7),8-diene	1005	1003 a	-	-	-	-	-	-	-	-	0.1	-
α-phellandrene	1005	1002 a	-	0.2	-	-	-	-	0.9	-	-	0.2
δ-3-carene	1011	1008 a	-	-	-	-	-	-	0.5	-	-	-
1,4-cineole	1015	1012 a	-	-	-	-	-	-	1.0	-	-	-
α-terpinene	1017	1017 b	-	10.3	-	-	-	0.1	1.4	-	-	1.2
p-menth-1-ene	1022	1021 a	-	0.2	-	-	-	-	-	-	-	-
p-cymene	1024	1020 a	-	7.2	0.6	0.3	13.1	2.1	17.0	1.2	20.5	13.5
limonene	1028	1024 a	-	3.6	0.6	8.5	30.6	-	-	8.8	3.5	1.0
eucalyptol	1030	1026 a	0.2	3.7	0.4	4.8	-	24.0	44.4	12.4	2.5	1.2
γ-terpinene	1058	1054 a	-	16.3	-	-	3.9	2.4	4.0	-	-	3.8
trans-furanolinalool oxide	1072	1072 b	-	0.4	0.1	-	1.0	-	-	-	-	-
cis-furanolinalool oxide	1088	1088 b	-	-	0.1	-	1.3	-	-	-	-	-
α-terpinolene	1089	1088 b	-	3.9	-	-	-	0.1	3.4	-	-	-
plinol a	1091	N/A	-	-	-	0.1	-	-	-	-	-	-
α-pinene epoxide	1098	1099 a	-	-	-	-	-	-	-	0.4	-	-
linalool	1102	1100 b	-	-	9.6	30.9	18.7	2.8	0.9	2.1	11.4	4.0
cis-rose oxide	1111	1111 b	-	-	1.2	-	-	-	-	-	-	-
trans-2-pinanol	1121	1119 a	-	-	-	0.2	-	-	-	-	-	-
3-pinanol	1122	1121 c	-	-	-	0.3	-	-	-	-	0.1	-
plinol D	1124	1125 b	-	-	-	0.7	-	-	-	-	0.2	-
trans-rose oxide	1127	1127 b	-	-	0.1	-	-	-	-	-	-	-
cis-limonene oxide	1133	1132 a	-	-	-	-	1.0	-	-	0.1	-	-
1-terpineol	1134	1130 a	-	-	-	0.2	-	-	-	-	-	-
trans-limonene oxide	1137	1137 a	-	-	-	-	0.6	-	-	-	-	-
trans-pinocarveol	1138	1135 a	-	0.3	-	-	-	-	-	-	-	-
camphor	1143	1141 a	-	-	-	6.9	-	21.9	-	22.1	0.2	0.1
cis-β-terpineol	1144	1140 a	-	0.4	0.2	-	-	-	-	-	-	-
epoxyterpinolene	1145	1145 b	-	-	-	-	0.2	-	0.3	-	-	-
menthone	1153	1154 b	-	-	2.4	-	-	-	-	-	-	-
trans-β-terpineol	1163	1159 a	-	-	-	0.2	-	-	-	-	-	-
iso-menthone	1164	1164 b	-	-	7.1	-	-	-	-	-	-	-
borneol	1164	1165 a	-	-	-	0.8	-	0.4	-	8.3	0.3	-
terpinen-4-ol	1176	1174 a	-	32.1	-	0.7	-	2.9	-	0.3	0.1	-
p-cymen-8-ol	1184	1186 b	-	-	-	-	-	-	0.5	-	-	-
α-terpineol	1190	1186 a	-	9.3	1.3	5.2	0.2	3.1	11.6	4.3	6.2	-
myrtenal	1195	1195 a	-	-	-	-	-	-	-	0.2	-	-
dihydrocitronellol	1196	1196 b	-	-	0.6	-	-	-	-	-	-	-
epoxylinalol	1196	1198 c	-	-	-	-	0.4	-	-	-	-	-
γ-terpineol	1196	1199 a	-	0.3	-	1.8	-	-	-	-	0.9	-
monoterpenoide	1203	-	-	0.3	-	-	-	-	-	-	-	-
citronellol	1230	1229 b	-	-	30.8	-	-	-	-	-	0.1	-
monoterpenoide acetate	1233	-	-	-	-	0.2	-	-	-	-	-	-
neral	1241	1242 b	-	-	0.7	-	-	-	-	-	-	-
carvone	1243	1239 a	-	-	-	-	-	-	-	0.1	-	-
plinol d	1248	N/A	-	-	-	0.1	-	-	-	-	-	-
monoterpenoide	1252	-	-	0.4	-	-	-	-	-	-	-	-
linalyl acetate	1257	1254 a	-	-	-	29.1	14.6	-	-	10.8	0.1	-
geraniol	1258	1255 b	-	-	15.6	-	-	-	-	-	-	-
monoterpenoide formate	1266	-	-	-	0.4	-	-	-	-	-	-	-
1,4-dihydroxy-p-menth-2-ene	1268	1264 c	-	-	-	-	-	-	0.3	-	-	-
geranial	1270	1272 b	-	-	1.2	-	-	-	-	-	-	-
citronellyl formate	1277	1277 b	-	-	10.6	-	-	-	-	-	-	-
neryl formate	1281	1280 a	-	-	1.7	-	-	-	-	-	-	-
iso-isopulegyl acetate	1283	1283 b	-	-	-	0.2	-	-	0.3	-	-	-
bornyl acetate	1285	1287 a	-	-	-	0.6	-	-	-	7.8	-	-
lavandulyl acetate	1290	1288 a	-	-	-	0.4	-	-	-	-	-	-
trans-sabinyl acetate	1292	1289 a	-	-	-	-	-	-	-	1.8	-	-
thymol	1292	1289 a	-	-	-	-	-	-	1.0	-	38.7	17.5
carvacrol	1297	1298 a	-	-	-	-	-	-	-	-	5.2	46.4
geranyl formate	1302	1303 b	-	-	5.1	-	-	-	-	-	-	-
(1R,4R)-p-mentha-2,8-diene, 1-hydroperoxide	1320	N/A	-	-	-	-	0.1	-	-	0.1	-	-
p-mentha-1,4,-dien-7-ol	1322	1325 a	-	-	-	-	-	-	-	0.2	-	-
citronellol epoxide	1331	N/A	-	-	0.5	-	-	-	-	-	-	-
α-terpinyl acetate	1348	1346 a	-	-	-	-	-	-	1.7	1.1	-	-
(2R,4R)-p-mentha-6,8-diene, 2-hydroperoxide	1362	1365 c	-	-	-	-	-	-	-	0.2	-	-
geranyl acetate	1382	1379 a	-	-	-	-	-	-	-	0.1	-	-
geranyl propanoate	1474	1476 a	-	-	0.7	-	-	-	-	0.2	-	-
geranyl isobutanoate	1514	1514 a	-	-	0.8	-	-	-	-	-	-	-
geranyl butanoate	1561	1562 a	-	-	1.1	-	-	-	-	-	-	-
geranyl tiglate	1701	1703 b	-	-	0.5	-	-	-	-	-	-	-
Sesquiterpenes:			21.6	6.8	3.2	7.1	-	5.1	1.0	1.7	4.3	5.8
β-bourbonene	1384	1387 a	-	-	0.4	-	-	-	-	-	-	-
(Z)-patchenol	1314	1316 a	-	-	-	-	-	-	-	0.3	-	-
α-gurjunene	1409	1409 a	-	0.4	-	-	-	-	-	-	-	-
β-caryophyllene	1419	1417 a	13.3	-	1.4	7.1	-	4.7	1.0	0.3	4.0	4.7
aromandendrene	1439	1439 b	-	4.5	-	-	-	-	-	-	-	-
(Z)-β-farnesene	1443	1444 a	-	-	0.1	-	-	-	-	-	-	-
selina-5,11-diene	1443	1442 b	-	0.2	-	-	-	-	-	-	-	-
α-humulene	1454	1452 a	5.1	-	-	-	-	0.3	-	0.1	-	-
β-selinene	1486	1489 a	-	0.1	-	-	-	-	-	-	-	-
allo-aromadendr-9-ene	1489	1489 b	-	0.2	-	-	-	-	-	-	-	-
viridiflorene	1496	1496 a	-	0.9	-	-	-	-	-	-	-	-
α-selinene	1496	1498 a	-	-	-	-	-	-	-	-	-	-
δ-cadinene	1524	1522 a	0.8	-	0.1	-	-	-	-	-	-	-
cyclocaryophyllane aldehyde	1553	1555 b	0.4	-	-	-	-	-	-	-	-	-
dihydrocaryophyllene-5-one	1559	1561 b	-	-	-	-	-	-	-	-	-	-
spathulenol	1578	1577 a	-	-	-	-	-	-	-	0.1	-	-
caryophyllene oxide	1583	1582 a	1.6	-	0.8	-	-	0.1	-	0.6	0.2	1.1
globulol	1585	1587 b	-	0.5	-	-	-	-	-	-	-	-
humulene-6,7-epoxide	1610	1612 b	0.3	-	-	-	-	-	-	0.1	-	-
10-epi-γ-eudesmol	1620	1622 a	-	-	0.4	-	-	-	-	-	-	-
Fenylopropanoids:			78.2	-	-							
eugenol	1359	1356 a	66.0	-	-	-	-	-	-	-	-	-
eugenol acetate	1529	1530 b	12.3	-	-	-	-	-	-	-	-	-
Other compounds:			-	-	1.1	-	-	-	-	-	-	-
6-methyl-5-hepten-2-one	986	986 b	-	-	0.3	-	-	-	-	-	-	-
phenyl ethyl alcohol	1113	1112 b	-	-	0.4	-	-	-	-	-	-	-
2-phenylethyl tiglate	1585	1584 a	-	-	0.4	-	-	-	-	-	-	-

N/A—not available. ^a^ —Adams [29]). ^b^ —Tkachev [30]. ^c^ —https://webbook.nist.gov (accessed on 1 March 2025).

**Table 2:** Descriptive statistics of absorbance values following treatment with different EOs and AMB at different concentrations.

Oil	Concentration	N	Average	SE	Median	SD	Min	Max	IQR
Control group	1%	14	0.541	0.030	0.530	0.113	0.395	0.704	0.205
	2%	14	0.515	0.019	0.496	0.071	0.406	0.631	0.108
AMB	0.5 mg/L	12	0.079	0.004	0.078	0.013	0.057	0.111	0.009
	1 mg/L	12	0.083	0.004	0.078	0.013	0.067	0.107	0.020
Bergamot oil	1%	14	0.354	0.054	0.358	0.201	0.137	0.562	0.397
	2%	14	0.265	0.034	0.264	0.129	0.127	0.400	0.245
Cajeput oil	1%	14	0.353	0.044	0.344	0.163	0.177	0.544	0.306
	2%	14	0.403	0.033	0.452	0.123	0.239	0.558	0.241
Clove oil	1%	14	0.852	0.022	0.831	0.081	0.728	0.959	0.137
	2%	14	0.886	0.015	0.911	0.056	0.789	0.963	0.098
Geranium oil	1%	14	0.362	0.041	0.334	0.152	0.193	0.547	0.299
	2%	14	0.287	0.029	0.292	0.108	0.163	0.405	0.211
Lavender oil	1%	14	0.309	0.042	0.349	0.156	0.129	0.467	0.306
	2%	14	0.237	0.028	0.213	0.105	0.125	0.358	0.206
Oregano oil	1%	14	0.315	0.047	0.356	0.176	0.143	0.788	0.226
	2%	14	0.289	0.030	0.282	0.113	0.171	0.420	0.215
Rosemary oil	1%	14	0.314	0.049	0.302	0.184	0.123	0.513	0.351
	2%	14	0.281	0.044	0.228	0.166	0.122	0.531	0.296
Sage oil	1%	14	0.340	0.051	0.319	0.190	0.144	0.557	0.376
	2%	14	0.325	0.038	0.324	0.142	0.176	0.476	0.271
Tea tree oil	1%	14	0.549	0.017	0.536	0.065	0.432	0.629	0.113
	2%	14	0.528	0.027	0.493	0.102	0.380	0.644	0.195
Thyme oil	1%	14	0.440	0.030	0.414	0.112	0.317	0.599	0.203
	2%	14	0.500	0.014	0.478	0.051	0.447	0.575	0.098

**Table 3:** The list of the tested EO with their assigned codes.

Group	Code
Clove oil	1
Tea tree oil	2
Geranium oil	3
Lavender oil	4
Bergamot oil	5
Rosemary oil	6
Cajeput oil	7
Sage oil	8
Thyme oil	9
Oregano oil	10
Control	11

**Table 4:** The *Prototheca* spp. strains isolated from Polish dairy herds, according to their location.

Strain	Species	Herd Location	Material	Preliminary Identification	GenBank
PRO3	*Prototheca bovis*	Kuyavian- Pomerania	Quarter milk	Phenotypic characteristics	PQ151373
PRO7	*Prototheca bovis*	West Pomerania	Quarter milk	Phenotypic characteristics	PQ151374

## Data Availability

The dataset is available upon request from the authors.

## References

[1] Jagielski T., Krukowski H., Bochniarz M., Piech T., Roeske K., Bakuła Z., Wlazło Ł., Woch P. (2019). Prevalence of *Prototheca* spp. on dairy farms in Poland—A cross-country study. Microb. Biotechnol..

[2] Kalińska A., Gołębiewski M., Wójcik A. (2017). Mastitis pathogens in dairy cattle—A review. World Sci. News.

[3] McDougall S., Castle R. (2021). Cow-level risk factors for clinical mastitis in the dry period in cows treated with an internal teat sealant alone at the end of lactation. N. Zeal. Vet. J..

[4] Heikkilä A.M., Nousiainen J.I., Pyörälä S. (2012). Costs of clinical mastitis with special reference to premature culling. J. Dairy Sci..

[5] Wawron W., Bochniarz M., Piech T. (2010). Yeast mastitis in dairy cows in the middle-eastern part of Poland. Bull. Vet. Inst. Pulawy.

[6] Park H.-S., Moon D.C., Hyun B.-H., Lim S.-K. (2019). Occurrence and persistence of *Prototheca zopfii* in dairy herds of Korea. J. Dairy Sci..

[7] Morandi S., Cremonesi P., Capra E., Silvetti T., Decimo M., Bianchini V., Alves A., Vargas A., Costa G., Ribeiro M. (2016). Molecular typing and differences in biofilm formation and antibiotic susceptibilities among *Prototheca* strains isolated in Italy and Brazil. J. Dairy Sci..

[8] Li J., Chen X., Jin E., Wang G., Wu L., Shao Z., Wan P., Hu C., Li J., Chen J. (2021). A survey of *Prototheca bovis* infection in dairy farms of the Hubei province China. J. Vet. Med. Sci..

[9] Toyotome T., Matsui S. (2022). Analysis of *Prototheca* and yeast species isolated from bulk tank milk collected in Tokachi District, Japan. J. Dairy Sci..

[10] Jagielski T., Buzzini P., Lassa H., Malinowski E., Branda E., Turchetti B., Polleichtner A., Roesler U., Lagneau P.E., Marques S. (2012). Multicentre Etest evaluation of in vitro activity of conventional antifungal drugs against European bovine Mastitis *prototheca* spp. isolates. J. Antimicrob. Chemother..

[11] Lerche M. (1952). Eine durch Algen (*Prototheca*) hervorgerufene Mastitis der Kuh. Berl. Munch. Tierarztl. Wochenschr..

[12] Bozzo G., Bonerba E., Di Pinto A., Bolzoni G., Ceci E., Mottola A., Tantillo G., Terio V. (2014). Occurrence of *Prototheca* spp. in cow milk samples. New Microbiol..

[13] Libisch B., Picot C., Ceballos-Garzon A., Moravkova M., Klimesová M., Telkes G., Chuang S.T., Le Pape P. (2022). *Prototheca* Infections and Ecology from a One Health Perspective. Microorganisms.

[14] Kano R., Kazuo Satoh Yaguchi T., Masuda M., Makimura K., de Hoog G.S. (2022). Phenotypic Characteristics of *Prototheca* Species Occurring in Humans and Animals. Med. Mycol. J..

[15] Mshana S.E., Sindato C., Matee M.I., Mboera L.E.G. (2021). Antimicrobial Use and Resistance in Agriculture and Food Production Systems in Africa: A Systematic Review. Antibiotics.

[16] Ifedinezi O.V., Nnaji N.D., Anumudu C.K., Ekwueme C.T., Uhegwu C.C., Ihenetu F.C., Obioha P., Simon B.O., Ezechukwu P.S., Onyeaka H. (2024). Environmental Antimicrobial Resistance: Implications for Food Safety and Public Health. Antibiotics.

[17] Lambraki I.A., Cousins M., Graells T., Léger A., Henriksson P., Harbarth S., Troell M., Wernli D., Søgaard Jørgensen P., Desbois A.P. (2022). Factors influencing antimicrobial resistance in the European food system and potential leverage points for intervention: A participatory, One Health study. PLoS ONE..

[18] Wińska K., Mączka W., Łyczko J., Grabarczyk M., Czubaszek A., Szumny A. (2019). Essential Oils as Antimicrobial Agents-Myth or Real Alternative?. Molecules.

[19] Burt S. (2004). Essential oils: Their antibacterial properties and potential applications in foods—A review. Int. J. Food Microbiol..

[20] Stringaro A., Colone M., Angiolella L. (2018). Antioxidant, Antifungal, Antibiofilm, and Cytotoxic Activities of *Mentha* spp. Essential Oils. Medicines.

[21] Nazzaro F., Fratianni F., De Martino L., Coppola R., De Feo V. (2013). Effect of Essential Oils on Pathogenic Bacteria. Pharmaceuticals.

[22] Alexa V.T., Galuscan A., Soica C.M., Cozma A., Coricovac D., Borcan F., Popescu I., Mioc A., Szuhanek C., Dehelean C.A. (2022). In Vitro Assessment of the Cytotoxic and Antiproliferative Profile of Natural Preparations Containing Bergamot, Orange and Clove Essential Oils. Molecules.

[23] Huang Y., Xu H., Ding M., Li J., Wang D., Li H., Sun M., Xia F., Bai H., Wang M. (2023). Screening of Rosemary Essential Oils with Different Phytochemicals for Antioxidant Capacity, Keratinocyte Cytotoxicity, and Anti-Proliferative Activity. Molecules.

[24] Bhattacharya R., Rolta R., Dev K., Sourirajan A. (2021). Synergistic potential of essential oils with antibiotics to combat fungal pathogens: Present status and future perspectives. Phytother. Res..

[25] Reichling J., Schnitzler P., Suschke U., Saller R. (2009). Essential oils of aromatic plants with antibacterial, antifungal, antiviral, and cytotoxic properties—An overview. Complement. Med. Res..

[26] Tariq S., Wani S., Rasool W., Shafi K., Bhat M.A., Prabhakar A., Shalla A.H., Rather M.A.A. (2019). Comprehensive review of the antibacterial, antifungal and antiviral potential of essential oils and their chemical constituents against drug-resistant microbial pathogens. Microb. Pathog..

[27] Bakkali F., Averbeck S., Averbeck D., Idaomar M. (2008). Biological effects of essential oils—A review. Food Chem. Toxicol..

[28] Wani A.R., Yadav K., Khursheed A., Rather M.A. (2021). An updated and comprehensive review of the antiviral potential of essential oils and their chemical constituents with special focus on their mechanism of action against various influenza and coronaviruses. Microb. Pathog..

[29] Adams R.P. (2007). Identification of Essential Oil Components by Gas Chromatography/Mass Spectrometry.

[30] Tkachev A.V. (2008). Investigation of Plant’s Volatile Compounds.

[31] Chaieb K., Hajlaoui H., Zmantar T., Kahla-Nakbi A.B., Rouabhia M., Mahdouani K., Bakhrouf A. (2007). The chemical composition and biological activity of clove essential oil, Eugenia caryophyllata (*Syzigium aromaticum* L. *Myrtaceae*): A short review. Phytother. Res..

[32] Kiki M.J. (2023). In vitro antiviral potential, antioxidant, and chemical composition of clove (*Syzygium aromaticum*) essential oil. Molecules.

[33] Carson C.F., Hammer K.A., Riley T.V. (2006). *Melaleuca alternifolia* (Tea Tree) oil: A review of antimicrobial and other medicinal properties. Clin. Microbiol. Rev..

[34] Hammer K.A. (2015). Treatment of acne with tea tree oil (melaleuca) products: A review of efficacy, tolerability and potential modes of action. Int. J. Antimicrob. Agents.

[35] Prabuseenivasan S., Jayakumar M., Ignacimuthu S. (2006). In vitro antibacterial activity of some plant essential oils. BMC Complement. Altern. Med..

[36] Senthil Kumar K.J., Gokila Vani M., Wang C.S., Chen C.C., Chen Y.C., Lu L.P., Huang C.H., Lai C.S., Wang S.Y. (2020). Geranium and lemon essential oils and their active compounds downregulate angiotensin-converting enzyme 2 (ACE2), a SARS-CoV-2 spike receptor-binding domain, in epithelial cells. Plants.

[37] Li Y., Liu S., Zhao C., Zhang Z., Nie D., Tang W., Li Y. (2022). The Chemical Composition and Antibacterial and Antioxidant Activities of Five Citrus Essential Oils. Molecules.

[38] Al-Sereiti M.R., Abu-Amer K.M., Sen P. (1999). Pharmacology of rosemary (*Rosmarinus officinalis* Linn.) and its therapeutic potentials. J. Exp. Biol..

[39] Retnosari S., Retnosari R., Asmaningrum H.P. (2018). Profile of the Indonesian essential oil from melaleuca cajuputi. Adv. Eng. Res..

[40] Sharifi-Rad M., Ozcelik B., Altı G., Daşkaya-Dikmen C., Martorell M., Ramírez-Alarcón K., Alarcón-Zapata P., Morais-Braga M.F.B., Carneiro J.N.P., Borges Leal A.L.A. (2018). Salvia spp. plants-from farm to food applications and phytopharmacotherapy. Trends Food Sci. Technol..

[41] De Martino L., De Feo V., Nazzaro F. (2009). Chemical composition and in vitro antimicrobial and mutagenic activities of seven Lamiaceae essential oils. Molecules.

[42] Teles A.M., Rosa T.D.D.S., Mouchrek A.N., Abreu-Silva A.L., Calabrese K.D.S., Almeida-Souza F. (2019). Cinnamomum zeylanicum, Origanum vulgare, and Curcuma longa Essential Oils: Chemical Composition, Antimicrobial and Antileishmanial Activity. Evid. Based Complement. Alternat. Med..

[43] Jagielski T., Gawor J., Bakuła Z., Decewicz P., Maciszewski K., Karnkowska A. (2018). Cytb as a New Genetic Marker for Differentiation of Prototheca Species. J. Clin. Microbiol..

[44] Grzesiak B., Głowacka A., Krukowski H., Lisowski A., Lassa H., Sienkiewicz M. (2016). The in vitro efficacy of essential oils and antifungal drugs against *Prototheca zopfii*. Mycopathologia.

[45] Grzesiak B., Kołodziej B., Głowacka A., Krukowski H. (2018). The effect of some natural essential oils against bovine *mastitis* caused by *Prototheca zopfii* isolates in vitro. Mycopathologia.

[46] Nardoni S., Pisseri F., Pistelli L., Najar B., Luini M., Mancianti F. (2018). In Vitro Activity of 30 Essential Oils against Bovine Clinical Isolates of *Prototheca zopfii* and *Prototheca blaschkeae*. Vet. Sci..

[47] Catoi C., Bolfă P.F., Tabaran F.A., Borza G. (2010). The inhibitory effect of some natural essential oils upon Prototheca algae in vitro growth. Bull. UASVM.

[48] Abbasi Sani B., Eidi S., Ghodrati Azadi H. (2023). In vitro activity of some native Iranian plants in Prototheca isolated from Clinical Bovine Mastitis. J. Vet. Lab. Res..

[49] Nojo H., Ishijima S.A., Morikawa M., Ito T., Kano R. (2024). In vitro susceptibility testing of phytochemicals from essential oils against Prototheca species. J. Vet. Med. Sci..

[50] Nelson R.R. (1997). In-vitro activities of five plant essential oils against methicillin-resistant Staphylococcus aureus and vancomycin-resistant Enterococcus faecium. J. Antimicrob, Chemother..

[51] Tardugno R., Serio A., Pellati F., D’Amato S., Chaves López C., Bellardi M.G., Di Vito M., Savini V., Paparella A., Benvenuti S. (2019). Lavandula x intermedia and Lavandula angustifolia essential oils: Phytochemical composition and antimicrobial activity against foodborne pathogens. Nat. Prod. Res..

[52] Bozin B., Mimica-Dukic N., Samojlik I., Jovin E. (2007). Antimicrobial and antioxidant properties of rosemary and sage (*Rosmarinus officinalis* L. and *Salvia officinalis* L., *Lamiaceae*) essential oils. J. Agric. Food Chem..

[53] Luqman S., Dwivedi G.R., Darokar M.P., Kalra A., Khanuja S.P. (2007). Potential of rosemary oil to be used in drug-resistant infections. Altern. Ther. Health Med..

[54] Adame-Gallegos J.R., Andrade-Ochoa S., Nevarez-Moorillon G.V. (2016). Potential use of Mexican oregano essential oil against parasite, fungal and bacterial pathogens. J. Essent. Oil-Bear. Plants.

[55] Rodriguez-Garcia I., Silva-Espinoza B.A., Ortega-Ramirez L.A., Leyva J.M., Siddiqui M.W., Cruz-Valenzuela M.R., Gonzalez-Aguilar G.A., Ayala-Zavala J.F. (2016). Oregano Essential Oil as an Antimicrobial and Antioxidant Additive in Food Products. Crit. Rev. Food Sci. Nutr..

[56] Massa N., Cantamessa S., Novello G., Ranzato E., Martinotti S., Pavan M., Rocchetti A., Berta G., Gamalero E., Bona E. (2018). Antifungal activity of essential oils against azole-resistant and azole-susceptible vaginal *Candida glabrata* strains. Can. J. Microbiol..

[57] Philpot W.N., Nickerson S.C. (1991). Mastitis: Counter Attack—A Strategy to Combat Mastitis.

[58] Stein S.E. (2020). NIST 2020 Mass Spectral Library.

[59] McLafferty F.W., Stauffer D.B. (2020). Wiley Science Solutions. Wiley Registry of Mass Spectral Data.

[60] Pore R.S. (1973). Selective medium for the isolation of Prototheca. Appl. Microbiol..

[61] Jagielski T., Bakuła Z., Gawor J., Maciszewski K., Kusber W., Dyląg M., Nowakowska J., Gromadka R., Karnkowska A. (2019). The genus *Prototheca* (Trebouxiophyceae, Chlorophyta) revisited: Implications from molecular taxonomic studies. Algal Res..

[62] Morello L., Tiroli T., Aretino F., Morandi S., Breviario D. (2020). Preliminary Results, Perspectives, and Proposal for a Screening Method of In Vitro Susceptibility of *Prototheca* Species to Antimicrotubular Agents. Antimicrob Agents Chemother.

[63] Dziurzyński M., Decewicz P., Iskra M., Bakuła Z., Jagielski T. (2021). Prototheca-ID: A web-based application for molecular identification of Prototheca species. Database.

[64] Betlej I., Andres B., Cebulak T., Kapusta I., Balawejder M., Jaworski S., Lange A., Kutwin M., Pisulewska E., Kidacka A. (2023). Antimicrobial Properties and Assessment of the Content of Bioactive Compounds *Lavandula angustifolia* Mill. Cultivated in Southern Poland. Molecules.

[65] Kalińska A., Wawryło C., Tlatlik W., Gołębiewski M., Kot M., Lange A., Jaworski S. (2024). Preliminary In Vitro Evaluation of Silver, Copper and Gold Nanoparticles as New Antimicrobials for Pathogens That Induce Bovine Locomotion Disorders. Int. J. Mol. Sci..

[66] Kalińska A., Jaworski S., Wierzbicki M., Kot M., Radzikowski D., Smulski S., Gołębiewski M. (2023). Silver and Copper Nanoparticles as the New Biocidal Agents Used in Pre- and Post-Milking Disinfectants with the Addition of Cosmetic Substrates in Dairy Cows. Int. J. Mol. Sci..

[67] Sandve G.K., Nekrutenko A., Taylor J., Hovig E. (2013). Ten simple rules for reproducible computational research. PLoS Comput. Biol..

[68] Peng R.D. (2011). Reproducible research in computational science. Science.

[69] Xie Y. (2015). Dynamic Documents with R and Knitr.

[70] R Core Team (2024). R: A Language and Environment for Statistical Computing.

[71] Kassambara A. (2023). ggpubr: ‘ggplot2′ Based Publication Ready Plots.

[72] Kassambara A., Mundt F. (2020). factoextra: Extract and Visualize the Results of Multivariate Data Analyses.

[73] Lê S., Josse J., Husson F. (2008). FactoMineR: A Package for Multivariate Analysis. J. Stat. Softw..

[74] Patil I. (2021). Visualizations with statistical details: The ‘ggstatsplot’ approach. J. Open Source Softw..

[75] Wickham H. (2016). ggplot2: Elegant Graphics for Data Analysis.

[76] Wickham H., Bryan J. (2023). readxl: Read Excel Files.

[77] Wickham H., François R., Henry L., Müller K., Vaughan D. (2023). dplyr: A Grammar of Data Manipulation.

